# Correlation between *PLCE1* rs2274223 variant and digestive tract cancer: A meta‐analysis

**DOI:** 10.1002/mgg3.589

**Published:** 2019-02-19

**Authors:** Qingfa Chen, Yu Wang, Yan Xu, Hai Lin, Fangxi Xue, Xingtian Chen

**Affiliations:** ^1^ Department of Gastroenterology Linyi Central Hospital Linyi China

**Keywords:** Digestive tract cancer, Genetic variant, Meta‐analysis, Phospholipase C ε‐1 (*PLCE1*)

## Abstract

**Background:**

The relationship between phospholipase C ε‐1 (*PLCE1*) rs2274223 variant and digestive tract cancer remains inconclusive despite extensive investigations. Therefore, we performed this meta‐analysis to obtain a more credible conclusion.

**Methods:**

PubMed, Medline, and Embase were systematic searched. Odds ratios (ORs) and 95% confidence intervals (CIs) were calculated.

**Results:**

A total of 27 studies were finally included. Pooled analyses suggested that *PLCE1* rs2274223 variant was significantly correlated with the likelihood of esophageal cancer (dominant model: *p *<* *0.001, OR = 0.77, 95% CI 0.72–0.83; recessive model: *p *<* *0.001, OR = 1.28, 95% CI 1.12–1.45; additive model: *p *<* *0.001, OR = 1.20, 95% CI 1.11–1.29; allele model: *p *<* *0.001, OR = 0.80, 95% CI 0.74–0.88) and gastric cancer (recessive model: *p *=* *0.001, OR = 1.27, 95% CI 1.10–1.47; allele model: *p *=* *0.03, OR = 0.88, 95% CI 0.78–0.98) in overall population. Further subgroup analyses showed that the positive results were mainly driven by the East Asians. However, no positive results were detected in Caucasians and West Asians.

**Conclusion:**

Our findings indicated that the *PLCE1* rs2274223 variant might serve as a promising genetic biomarker of esophageal and gastric cancer in East Asians.

## 1 INTRODUCTION

Digestive tract cancer refers to malignancy that occurs in the digestive tract. Commonly seen digestive tract cancer like esophageal cancer, gastric cancer, and colorectal cancer are leading causes of cancer‐related morbidity and mortality worldwide (Siegel, Ma, Zou, & Jemal, 2014). Despite rapid advances in early diagnosis and surgical treatment over the past few decades, the incidence of digestive tract cancer is still increasing, and it is estimated that over twenty percent of cancer‐related deaths are caused by digestive tract cancer (Ferlay et al., 2015). To date, the exact pathogenic mechanism of digestive tract cancer remains unknown. Although smoking, excessive alcohol intake, high consumption of red meat, and chronic viral infection were already identified as potential pathogenic factors of digestive tract cancer (El‐Zimaity et al., 2018), the fact that a great inter‐individual variability in disease susceptibility existed in these exposed to above‐mentioned carcinogenic factors indicated that genetic background is also vital for the development of digestive tract cancer.

Phospholipase C ε‐1 (PLCE1) cleaves phosphatidylinositol‐4,5‐bisphosphate to generate inositol 1,4,5‐ triphosphate (IP3) and diacylglycerol (DAG), two key second messengers that play vital roles in activating a cascade of intracellular responses that are responsible for regulating cell growth and differentiation (Zhao et al., 2014). Previous experimental studies showed that an elevated IP3 level was associated with suppression of p53, a crucial tumor suppressor gene (Li et al., 2014; Luo, 2014). Consequently, it is speculated that functional *PLCE1* polymorphisms may be implicated in the pathogenesis of multiple malignant disorders.

The rs2274223 variant is located on chromosome 10q23, the G to A substitution at this locus was correlated with amino acid change and enzymatic function alteration. So far, some pilot studies were already conducted to investigate possible correlations between *PLCE1* rs2274223 variant and the likelihood of digestive tract cancer. But the results of these studies were controversial, especially when they were conducted in different ethnicities (Ezgi, Merve, Hakan, & Gül, 2016; Palmer et al., 2012; Yang et al., 2012). Therefore, we conducted this meta‐analysis to better analyze the roles of *PLCE1* rs2274223 variant in digestive tract cancer.

## 2 MATERIALS AND METHODS

### 2.1 Literature search and inclusion criteria

The current meta‐analysis complied with the Preferred Reporting Items for Systematic Reviews and Meta‐analyses (PRISMA) statement (Moher, Liberati, Tetzlaff, & Altman, 2009). We searched PubMed, Medline, and Embase for potentially related articles that were published prior to November 2018 using the following key words: “Phospholipase C epsilon 1”, “PLCE1”, “polymorphism”, “variant”, “mutation”, “genotype”, “allele”, “gastric”, “stomach”, “esophageal”, “esophagus”, “colonrectal”, “rectal”, “colonal”, “colon”, “rectum”, “tumor”, “cancer”, “carcinoma”, “malignancy”, and “neoplasm”. We also screened the reference lists of all retrieved publications to identify other potentially relevant articles.

To test the research hypothesis of this meta‐analysis, included studies should meet all the following criteria: (a) case–control study on correlations between *PLCE1* rs2274223 variant and the likelihood of digestive tract cancer; (b) provide adequate data to calculate odds ratios (ORs) and 95% confidence intervals (CIs); (c) full text in English available. Studies were excluded if one of the following criteria was fulfilled: (a) not relevant to *PLCE1* rs2274223 variant and digestive tract cancer; (b) family‐based association studies; (c) case reports or case series; (d) abstracts, reviews, comments, letters, and conference presentations. For duplicate reports, only the study with the largest sample size was included.

### 2.2 Data extraction and quality assessment

The following data were extracted from all included studies: (a) the first author; (b) year of publication; (c) country and ethnicity of study subjects; (d) sample size; and (e) the distribution of *PLCE1* rs2274223 variant in cases and controls. Moreover, the probability value (*p* value) of Hardy–Weinberg equilibrium (HWE) was also calculated based on genotypic frequency of *PLCE1* rs2274223 variant in the control group.

The Newcastle–Ottawa scale (NOS) was used to assess the quality of eligible studies (Stang, 2010). The NOS has a score range of zero to nine, and studies with a score of more than seven were thought to be of high quality.

Two reviewers conducted data extraction and quality assessment independently. When necessary, the reviewers wrote to the corresponding authors for extra information or raw data. Any disagreement between two reviewers was solved by discussion until a consensus was reached.

### 2.3 Statistical analyses

Review Manager Version 5.3.3 (The Cochrane Collaboration, Software Update, Oxford, United Kingdom) was used for statistical analyses. We calculated ORs and 95% CIs to estimate associations of *PLCE1* rs2274223 variant with the likelihood of digestive tract cancer in dominant (AA vs. AG + GG), recessive (GG vs. AA + AG), additive (AG vs. AA + GG) and allele (A vs. G) models, and a *p* value of 0.05 or less was considered to be statistically significant. Between‐study heterogeneities were assessed by I^2^ statistic. If I^2^ was greater than 50 percent, random‐effect models (REMs) would be used for analyses on account of obvious between‐study heterogeneities. Otherwise, fixed‐effect models (FEMs) would be applied for analyses. We conducted further subgroup analyses by ethnicity of participants. We carried out sensitivity analyses to test the stability of synthetic results. We used funnel plots to evaluate possible publication biases.

## 3 RESULTS

### 3.1 Characteristics of included studies

**Figure 1:**
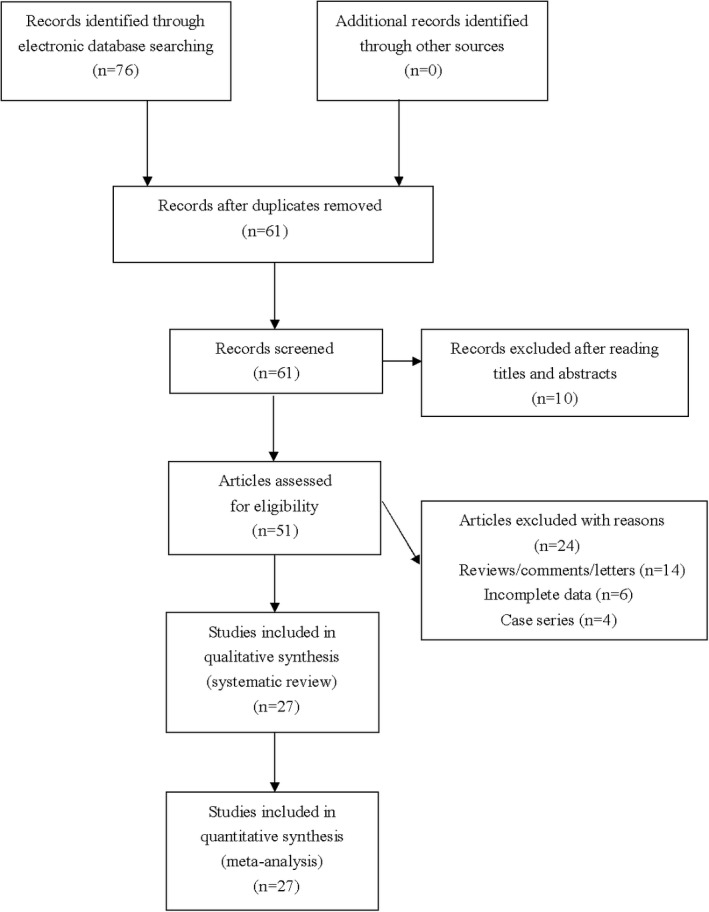
Flowchart of study selection for the present study

Totally 76 results were generated using our searching strategy. After reading titles and abstracts, 25 irrelevant and duplicate articles were excluded. Another 24 articles were subsequently excluded after reading the full text. Finally, a total of 27 eligible studies were included in the present meta‐analysis (see Figure 1). All eligible studies were published in English. The NOS score of eligible articles ranged from 7 to 8, which suggested that all included studies were of relatively high quality. Characteristics of included studies are shown in Table 1.

**Table 1:** The characteristics of included studies for *PLCE1 rs2274223* variant and digestive tract cancer

First author, year	Country	Ethnicity	Sample size	Genotype distribution		*P*‐value for HWE	NOS score
				Cases	Controls		
Esophageal cancer							
Abnet (2010)	USA	Mixed	2115/3302	NA	NA	NA	7
Bye (2012)	South Africa	Africa	672/1707	218/338/116	612/819/276	0.943	8
Chen (2013)	China	East Asian	200/300	97/84/19	178/111/11	0.211	8
Cui (2013)	China	East Asian	222/326	NA	NA	NA	7
Duan (2013)	China	East Asian	381/420	193/150/38	281/123/16	0.582	8
Dura (2013)	The Netherlands	Caucasian	344/580	160/154/30	279/247/54	0.950	8
Gu (2012)	China	East Asian	379/371	202/147/30	233/119/19	0.457	8
Hu (2012)	China	East Asian	1061/1211	594/400/67	754/399/58	0.577	8
Jia (2015)	China	East Asian	358/305	194/140/24	190/104/11	0.482	8
Malik (2014)	India	West Asian	135/195	65/58/12	100/78/17	0.748	8
Palmer et al. (2012)	USA	Caucasian	306/210	132/150/24	86/107/17	0.039	8
Palmer et al. (2012)	Poland	Caucasian	289/376	107/138/44	154/166/56	0.307	8
Piao (2014)	Korea	East Asian	322/1700	153/140/29	909/684/107	0.148	8
Umar (2014)	India	West Asian	293/314	162/120/11	168/127/19	0.436	8
Yang (2014)	China	East Asian	313/314	172/122/19	209/96/9	0.609	8
Zhou (2012)	China	East Asian	517/510	248/227/42	291/191/28	0.646	8
Gastric cancer							
Abnet (2010)	USA	Mixed	2240/3302	NA	NA	NA	7
Kupcinskas (2014)	Lithuania	Caucasian	250/241	94/126/30	91/116/34	0.760	8
Malik (2014)	India	West Asian	108/195	54/45/9	100/78/17	0.748	8
Palmer et al. (2012)	USA	Caucasian	159/210	74/68/17	86/107/17	0.039	8
Song (2014)	Korea	Mixed	3245/1700	1818/1197/230	909/684/107	0.148	8
Sun (2015)	China	East Asian	692/774	405/254/33	514/226/34	0.155	8
Wang (2012)	China	East Asian	1059/1240	600/399/60	791/390/59	0.224	8
Yang et al. (2012)	China	East Asian	249/292	NA	NA	NA	7
Yuan (2016)	China	East Asian	116/102	NA	NA	NA	7
Zhang (2011)	China	East Asian	1665/1848	867/664/134	1122/643/83	0.451	8
Colorectal cancer							
Ezgi et al. (2016)	Turkey	Caucasian	200/230	142/48/10	176/54/0	0.044	8
Kupcinskas (2014)	Lithuania	Caucasian	192/376	77/91/24	147/173/56	0.662	8
Li (2012)	China	East Asian	231/292	155/71/5	180/92/20	0.089	8
Wang (2014)	China	East Asian	417/416	NA	NA	NA	8

PLCE1: Phospholipase C‐epsilon 1; HWE: Hardy–Weinberg equilibrium; NOS: Newcastle–Ottawa scale; NA, Not available.

### 3.2 Overall and subgroup analyses

Among included studies for *PLCE1* rs2274223 variant and digestive tract cancer, fifteen studies were about esophageal cancer (7,907 cases and 12,141 controls), ten studies were about gastric cancer (9,783 cases and 9,904 controls) and four studies were about colorectal cancer (1,040 cases and 1,314 controls). Pooled analyses suggested that *PLCE1* rs2274223 variant was significantly correlated with the likelihood of esophageal cancer (dominant model: *p *<* *0.001, OR = 0.77, 95% CI 0.72–0.83; recessive model: *p *<* *0.001, OR = 1.28, 95% CI 1.12–1.45; additive model: *p *<* *0.001, OR = 1.20, 95% CI 1.11–1.29; allele model: *p *<* *0.001, OR = 0.80, 95% CI 0.74–0.88) and gastric cancer (recessive model: *p *=* *0.001, OR = 1.27, 95% CI 1.10–1.47; allele model: *p *=* *0.03, OR = 0.88, 95% CI 0.78–0.98) in overall population. Further subgroup analyses by ethnicity of participants revealed that the positive results were mainly driven by the East Asians. However, no positive results were detected in Caucasians and West Asians (see Table 2).

**Table 2:** Results of overall and subgroup analyses for *PLCE1 rs2274223* variant and digestive tract cancer

Population	Sample size	Dominant comparison			Recessive comparison			Additive comparison			Allele comparison		
		*p* value	OR (95% CI)	*I* ^2^ statistic	*p* value	OR (95% CI)	I^2^ statistic	*p* value	OR (95% CI)	*I* ^2^ statistic	*p* value	OR (95% CI)	*I* ^2^ statistic
Esophageal cancer													
Overall	7907/12141	**<0.001**	**0.77 (0.72–0.83)**	45%	**<0.001**	**1.28 (1.12–1.45)**	46%	**<0.001**	**1.20 (1.11–1.29)**	0%	**<0.001**	**0.80 (0.74–0.88)**	64%
Caucasian	939/1166	0.52	0.94 (0.79**–**1.13)	0%	0.88	0.98 (0.74**–**1.30)	0%	0.46	1.07 (0.90**–**1.27)	0%	0.68	0.97 (0.85**–**1.11)	0%
East Asian	3431/3757	**<0.001**	**0.68 (0.62–0.75)**	0%	**<0.001**	**1.74 (1.41–2.14)**	8%	**<0.001**	**1.31 (1.19–1.45)**	0%	**<0.001**	**0.71 (0.66–0.77)**	38%
West Asian	428/509	0.98	1.00 (0.78**–**1.30)	0%	0.37	0.78 (0.45**–**1.34)	0%	0.67	1.06 (0.81**–**1.37)	0%	0.71	1.04 (0.85**–**1.28)	0%
Gastric cancer													
Overall	9783/9904	0.19	0.88 (0.72**–**1.07)	83%	**0.001**	**1.27 (1.10–1.47)**	47%	0.30	1.10 (0.92**–**1.33)	81%	**0.03**	**0.88 (0.78–0.98)**	79%
Caucasian	409/451	0.50	1.10 (0.84**–**1.45)	0%	0.96	0.99 (0.65**–**1.51)	17%	0.62	0.90 (0.60**–**1.36)	56%	0.61	1.05 (0.86**–**1.29)	0%
East Asian	3781/4256	**<0.001**	**0.72 (0.65–0.79)**	0%	0.06	1.40 (0.99–1.98)	62%	**<0.001**	**1.30 (1.18–1.43)**	0%	**<0.001**	**0.77 (0.72–0.83)**	18%
Colorectal cancer													
Overall	1040/1314	0.48	0.89 (0.65**–**1.22)	68%	0.98	0.98 (0.23**–**4.09)	79%	0.88	1.02 (0.82–1.27)	0%	0.98	1.00 (0.69–1.47)	78%
Caucasian	392/606	0.52	0.91 (0.69**–**1.20)	25%	0.49	3.51 (0.10**–**23.03)	84%	0.75	1.05 (0.80–1.38)	0%	0.53	0.85 (0.52–1.47)	78%

The values in bold represent there is statistically significant differences between cases and controls.

OR: Odds ratio; CI: Confidence interval; NA, Not available; PLCE1: Phospholipase C‐epsilon 1.

### 3.3 Sensitivity analyses

We carried out sensitivity analyses to examine the stability of synthetic results by eliminating studies that violated HWE. No changes of results were observed in any comparisons, which indicated that our findings were statistically reliable.

### 3.4 Publication biases

We used funnel plots to evaluate potential publication biases. No obvious asymmetry of funnel plots was observed in any comparisons, which suggested that our findings were unlikely to be influenced by severe publication bias.

## 4 DISCUSSION

To the best of our knowledge, this is so far the most comprehensive meta‐analysis on correlations between *PLCE1* rs2274223 variant and digestive tract cancer, and our pooled analyses showed that the *PLCE1* rs2274223 variant may serve as a genetic biomarker of esophageal cancer and gastric cancer in East Asians. As for evaluation of heterogeneities, altogether obvious between‐study heterogeneities were detected in several comparisons, a reduction tendency of heterogeneity was found for East Asian and Caucasian subgroups in further stratified analyses, which suggested that differences in ethnic background could partially explain observed heterogeneities between studies.

There are several points that need to be pointed out about the current study. First, previous experimental studies revealed that the *PLCE1* rs2274223 variant was correlated with an Arg‐to‐His change of PLCE1 protein, which may result in abnormal enzymatic activity and give rise to the development of multiple malignancies including digestive tract cancer (Ezgi et al., 2016; Palmer et al., 2012; Yang et al., 2012). The positive findings of our meta‐analysis may partially owing to the functional significance of rs2274223 variant. Second, the pathogenic mechanism of digestive tract cancer is highly complex, and hence it is unlikely that a single genetic variant could significantly contribute to its development. Therefore, to better illustrate potential correlations of certain genetic variant with digestive tract cancer, we strongly recommend further studies to perform haplotype analyses and explore potential gene–gene interactions.

As with all meta‐analysis, this study certainly has some limitations. First, our results were derived from unadjusted analyses due to lack of raw data, and failure to conduct further adjusted analyses for age, gender, and co‐morbidity conditions may impact the reliability of our findings (Xie, Shi, & Liu, 2017). Second, obvious heterogeneities were detected in certain subgroup comparisons, which indicated that the inconsistent results of included studies could not be fully explained by differences in ethnic background, and other unmeasured characteristics of participants may also attribute to between‐study heterogeneities (Shi, Xie, Jia, & Li, 2016). Third, associations between *PLCE1* rs2274223 variant and digestive tract cancer may also be influenced by gene–gene and gene–environmental interactions. However, the majority of studies did not consider these potential interactions, which impeded us to perform relevant analyses accordingly (Zhu, Zheng, & Hu, 2018). Taken these limitations into consideration, the results of the current study should be interpreted with caution.

## 5 CONCLUSIONS

Overall, our meta‐analysis suggested that the *PLCE1* rs2274223 variant might serve as a potential biological marker of esophageal and gastric cancer in East Asians. However, further well‐designed studies are warranted to confirm our findings. Moreover, future investigations are needed to explore potential roles of *PLCE1* rs2274223 variant in the development of other types of cancer.

## CONFLICT OF INTEREST

The authors declare that they have no conflict of interest.

## ETHICAL APPROVAL

This article does not contain any studies with human participants or animals performed by any of the authors.

## INFORMED CONSENT

For this type of study formal consent is not required.
